# Intracranial arteriosclerosis and the risk of dementia: A population‐based cohort study

**DOI:** 10.1002/alz.13496

**Published:** 2023-10-09

**Authors:** Tim C. van den Beukel, Frank J. Wolters, Uwe Siebert, Wilko Spiering, M. Arfan Ikram, Meike W. Vernooij, Pim A. de Jong, Daniel Bos

**Affiliations:** ^1^ Department of Epidemiology Erasmus Medical Center Rotterdam CA The Netherlands; ^2^ Department of Radiology and Nuclear Medicine Erasmus Medical Center Rotterdam CA The Netherlands; ^3^ Department of Radiology and Nuclear Medicine University Medical Center Utrecht Utrecht GA The Netherlands; ^4^ Department of Neurology Erasmus Medical Center Rotterdam CA The Netherlands; ^5^ Alzheimer Center Erasmus Medical Center Rotterdam CA The Netherlands; ^6^ Center for Health Decision Science, Departments of Epidemiology and Health Policy & Management Harvard T.H. Chan School of Public Health, Boston Boston Massachusetts USA; ^7^ Institute of Public Health, Medical Decision Making and Health Technology Assessment, Department of Public Health, Health Services Research and Health Technology Assessment UMIT TIROL ‐ University for Health Sciences and Technology Austria; ^8^ Program on Cardiovascular Research, Institute for Technology Assessment and Department of Radiology, Massachusetts General Hospital Harvard Medical School, Boston Boston Massachusetts USA; ^9^ Department of Vascular Medicine University Medical Center Utrecht Utrecht GA The Netherlands; ^10^ Department of Epidemiology Harvard T.H. Chan School of Public Health Boston Massachusetts USA

**Keywords:** arterial calcification, arteriosclerosis, cohort study, dementia, epidemiology

## Abstract

**BACKGROUND:**

The impact of intracranial arteriosclerosis on dementia remains largely unclear.

**METHODS:**

In 2339 stroke‐free and dementia‐free participants (52.2% women, mean age 69.5 years) from the general population, we assessed intracranial carotid artery calcification (ICAC) and vertebrobasilar artery calcification (VBAC) as proxy for arteriosclerosis. Associations with dementia were assessed using Cox models. In addition, indirect effects through cerebral small vessel disease (cSVD) and subcortical brain structure volumes were assessed using causal mediation analyses.

**RESULTS:**

During a median of 13.4 years (25th–75th percentiles 9.9–14.5) of follow‐up, 282 participants developed dementia. Both ICAC presence (hazard ratio [HR]: 1.53, 95% confidence interval [CI]: 1.00–2.32]) and volume (HR per standard deviation: 1.19, 95% CI: 1.01–1.40) increased dementia risk. For VBAC, severe calcifications increased dementia risk (HR for third vs first volume tertile: 1.89, 95% CI: 1.00–3.59). These effects were mediated partly through increased cSVD (percentage mediated for ICAC: 13% and VBAC: 24%).

**DISCUSSION:**

Intracranial arteriosclerosis increases the risk of dementia.

## 1 BACKGROUND

Vascular disease is an important contributor to dementia, with modifiable cardiometabolic risk factors accounting for ≈25% of dementia incidence.^1^ Within this context, an increasing body of evidence suggests that intracranial arteriosclerosis, the hardening of arteries due to calcification, might play a role in the etiology of dementia and Alzheimer's disease (AD).^2^, ^3^, ^4^, ^5^ However, several important knowledge gaps remain to be addressed. First, prospective data on the impact of intracranial arteriosclerosis on dementia remain scarce, with the available evidence being based on relatively short follow‐up durations.^2^, ^3^ As both arteriosclerosis and dementia develop over the course of years, there is a need for longitudinal investigations covering extensive time intervals. Second, previous investigations have focused primarily on the anterior cerebral circulation, whereas the posterior circulation represents the basis of the vascularization of subcortical brain structures such as the thalamus and hippocampus, which are important in dementia etiology.^6^, ^7^, ^8^ Posterior cerebral arteriosclerosis is thought, at least in part, to be a separate entity from anterior arteriosclerosis, given that both have distinct cardiovascular risk profiles and markedly different prevalence rates.^9^ Moreover, arteriosclerosis of the posterior cerebral circulation is related to white matter pathology,^10^ and white matter pathology in dementia occurs more frequently in brain areas supplied by the posterior cerebral circulation (i.e., parieto‐occipital and posterior periventricular areas).^11^ Thus it is conceivable that arteriosclerosis of the posterior cerebral circulation might impact dementia risk. Third, the impact of distinct subtypes of intracranial arteriosclerosis on dementia remains elusive. The subtypes differ in morphology (the atherosclerotic subtype is characterized by thick calcified atherosclerotic plaque located in the arterial intima, and the internal elastic lamina [IEL] subtype is composed of long, circular calcifications of the IEL),^12^, ^13^, ^14^ occur independently on histology,^12^ and are thought to impair arterial functioning in different ways.^15^, ^16^ The independent contribution of each subtype of intracranial arteriosclerosis was demonstrated previously for stroke risk.^17^ Fourth, intracranial arteriosclerosis is a risk factor for stroke and cerebral small vessel disease (cSVD), which in turn represent important risk factors for dementia.^18^, ^19^, ^20^ Moreover, because the amygdala is vascularized predominantly by the anterior cerebral circulation and the thalamus and hippocampus by the posterior brain circulation, including subcortical brain structures might provide important insights into the location‐specific effects of intracranial arteriosclerosis on dementia.^6^, ^7^ Hence, stroke, cSVD, and subcortical structures might act as mediators on the pathway from arteriosclerosis to dementia, but it remains undetermined what share of dementia risk is mediated through these processes.

Given that several therapeutic options are under investigation that target arteriosclerosis, further elucidating the association between intracranial arteriosclerosis and dementia might in the future open up important novel avenues for dementia prevention.^21^ Therefore, we assessed the association of intracranial arteriosclerosis with dementia in a large, population‐based cohort with over a decade of follow‐up time.

## 2 METHODS

### 2.1 Study design and setting

The Rotterdam Study is an ongoing, prospective, population‐based cohort study aimed at investigating the determinants of chronic diseases in the middle‐aged and elderly. At study entry and every 3 to 4 years, all participants are reexamined in a dedicated research center. The design and protocol have been described in detail previously.^22^

**FIGURE 1 alz13496-fig-0001:**
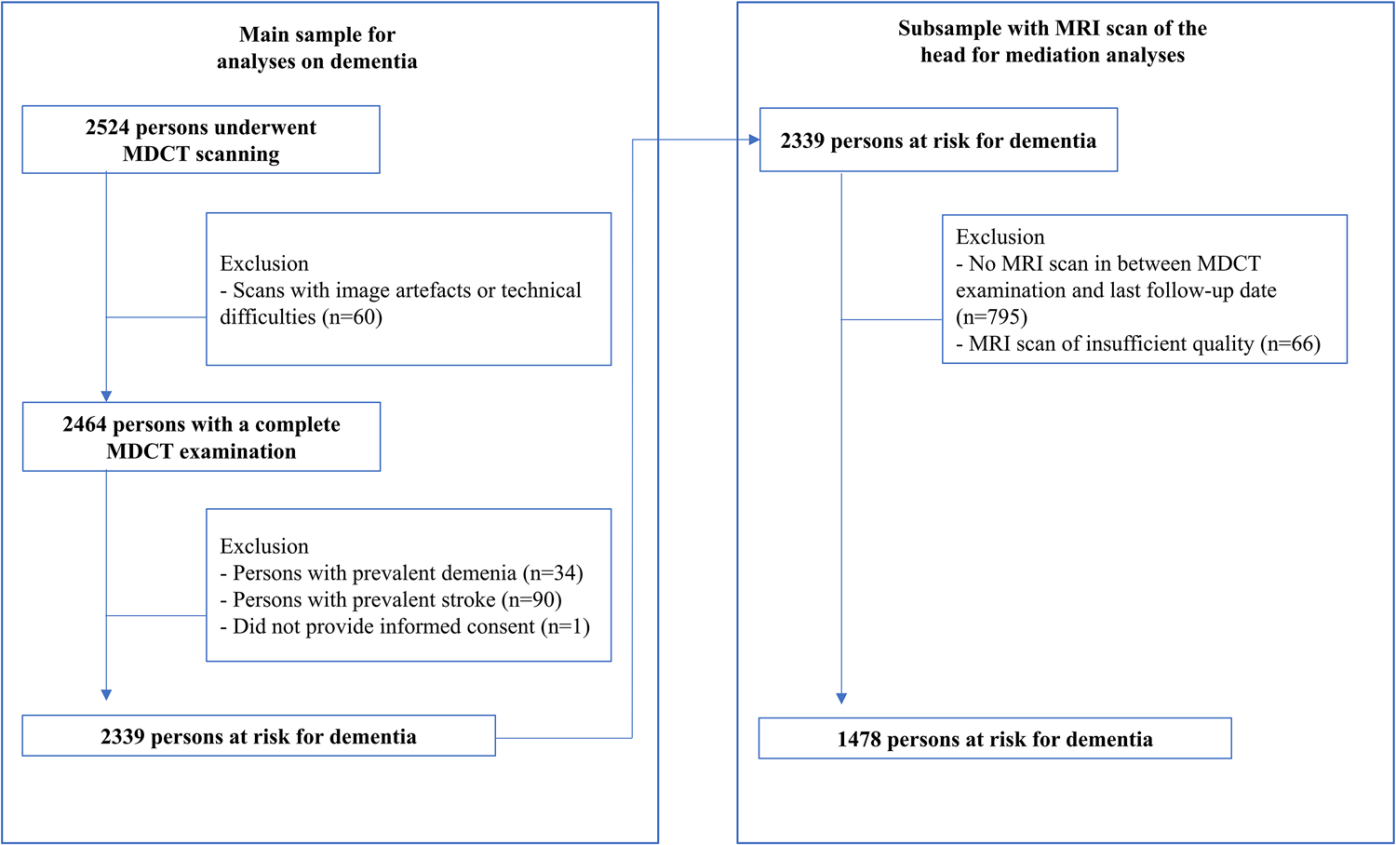
Flowchart of how the study samples were derived. MDCT, multidetector computed tomography; MRI, magnetic resonance imaging.

At the study visits between 2003 and 2006, a random selection of participants were invited to undergo a multidetector computed tomography (MDCT) scan. In total, 2524 participants were scanned (response rate: 78%). From 2005 onwards all participants were invited to undergo magnetic resonance imaging (MRI) of the brain. The current study focuses on all 2524 participants who underwent MDCT imaging. We excluded participants with image artifacts or technical difficulties (*n* = 60), prevalent dementia (n=34), prevalent stroke (*n* = 90), and those who did not provide informed consent for use of follow‐up data (*n* = 1, see Figure 1 for a flowchart). This left 2339 participants in the main sample for dementia risk analyses. For causal mediation analyses involving MRI‐based measurements, we further excluded participants who had not undergone an MRI of the head before their censor date (*n* = 795) and those with MRI scans of insufficient quality (*n* = 66), leaving 1478 persons at risk for dementia in this subsample for causal mediation analyses.

All participants were followed until the date of incident dementia, death, loss to follow‐up, or January 1, 2020, whichever came first. Follow‐up for dementia was complete for 98% of potential person‐years. The Rotterdam Study has been approved by the medical ethics review committee of the Erasmus Medical Center in Rotterdam (number MEC02.1015) and has been entered into the World Health Organization (WHO) International Clinical Trials Registry Platform (number NTR6831). All participants provided written informed consent for participation.

Requests to access the data may be sent to the management team of the Rotterdam Study (secretariat.epi@erasmusmc.nl), which has a protocol for approving data requests. Because of restrictions based on privacy regulations and informed consent of the participants, data cannot be made freely available in a public repository.

### 2.2 Assessment of intracranial arteriosclerosis

Intracranial arteriosclerosis was visualized on non‐enhanced MDCT images obtained from 16‐slice (*n* = 700) or 64‐slice (*n* = 1639) scanners (Somatom Sensation 16/64, Siemens, Forchheim, Germany) at 120 kVp and with a slice thickness of 1 mm. Further details on the scanning protocol have been reported previously.^23^

On these scans, we assessed intracranial carotid artery calcification (ICAC) and vertebrobasilar artery calcification (VBAC) as proxy for anterior and posterior intracranial arteriosclerosis respectively. Calcifications were assessed using a custom‐made plugin for ImageJ, in which regions of interest were drawn around calcifications in the arterial wall on consecutive MDCT scan slices by three trained raters. Calcification volume was calculated by multiplying the number of pixels above 130 Hounsfield units by pixel‐size and the increment. All volumes are expressed as cubic millimeters (mm^3^).^24^ The reproducibility of these ICAC and VBAC scores has previously been reported to be excellent (intraclass correlation coefficient for ICAC was 0.99^25^ and for VBAC was 0.99^26^).

**FIGURE 2 alz13496-fig-0002:**
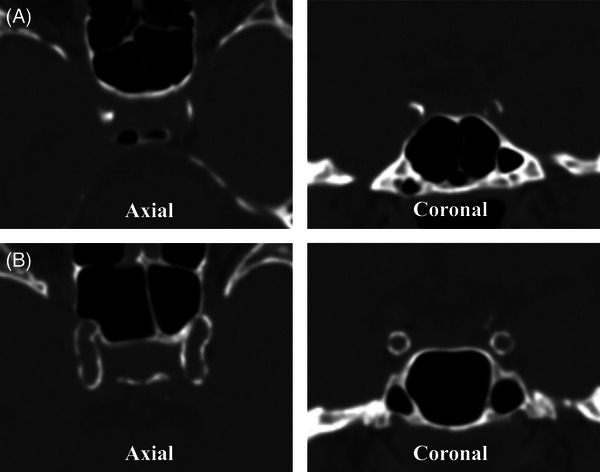
Illustrative computed tomography images of the atherosclerotic (A) and internal elastic lamina (B) subtypes of intracranial arteriosclerosis. The atherosclerotic subtype consists of thick, non‐circular calcifications, whereas the internal elastic lamina subtype is characterized by elongated, round calcification.

In addition, we determined the subtype of ICAC using a score that has been validated against histology.^27^ This score assigns points for calcification morphology, based on which a decision is made whether calcification is of the predominant atherosclerotic subtype (<7 points; non‐circular, thick, short calcifications) or IEL subtype (≥7 points; circular, thin, long calcifications).^27^ Figure 2 shows characteristic examples of each subtype of calcification. This score was applied to the left artery and right artery separately. We then classified persons as having the atherosclerotic subtype if they had bilateral atherosclerotic ICAC or if they had atherosclerotic ICAC in combination with no contralateral calcifications. The same method was used for classifying the IEL subtype. Participants with different subtypes in the left artery and right artery were classified as having a mixed ICAC subtype. The reproducibility of ICAC subtype scoring was reported previously to be good (proportion of agreement 93.9%, Cohen's kappa 0.88).^17^

**RESEARCH IN CONTEXT**

1. **Systematic review**: We performed a systematic search in Medline and identified 11 relevant articles. Two longitudinal studies reported mixed results, with the largest reporting a trend toward an increased risk of dementia associated with intracranial arteriosclerosis. No longitudinal studies included measurements of arteriosclerotic subtypes (internal elastic lamina [IEL] or atherosclerotic) or of anterior versus posterior cerebral arteriosclerosis.
2. **Interpretation**: Our findings led to a better understanding of dementia etiology. We found that arteriosclerosis of both the anterior and the posterior cerebral circulation increased the risk of dementia and Alzheimer's disease specifically. For anterior cerebral arteriosclerosis, especially the IEL subtype conferred dementia risk. Only parts of these effects were mediated through increased white matter hyperintensities, and not through stroke or subcortical brain structure volumes.
3. **Future directions**: Our findings, emphasize the important deleterious impact of intracranial arteriosclerosis on brain health, which was previously also demonstrated for stroke. Given the ubiquity of intracranial arteriosclerosis, it has considerable potential as a novel avenue for dementia prevention.

To determine ICAC subtype volume, we assessed ICAC volume in persons with a particular subtype, which was deemed appropriate as the occurrence of subtypes is uncorrelated on histology.^12^, ^13^ The subtype of VBAC was not determined, as the subtype scoring method has not been validated for VBAC.

### 2.3 Assessment of dementia

Participants were screened for dementia at entry into the Rotterdam Study and during follow‐up visits according to previously described protocol.^28^ In short, participants were screened with the Mini‐Mental State Examination (MMSE; a standardized test with a maximum score of 30, with lower scores indicating more severe cognitive problems) and the Geriatric Mental Schedule (GMS; a standardized, semi‐structured interview that results in a score from 0 through 5 on eight different symptom clusters, with higher scores indicating more symptoms). The eight symptom clusters are organicity (dementia and other organic brain syndromes), schizophrenia (and related psychoses), mania, neurotic depression, psychotic depression, hypochondriasis, phobias, obsessional neurosis, and anxiety neurosis).^29^, ^30^ Persons with an MMSE score of <26^29^ and/or a GMS‐organicity score >0^30^ underwent additional examination and informant interview with the Cambridge Examination for Mental Disorders in the Elderly. In addition, all participants were monitored for dementia through linkage of our study database with their medical records, including information from general practitioners, hospitals, nursing homes, and the regional institute for outpatient mental health care. Neuroimaging information was also retrieved if available. For all suspected cases of dementia, a consensus panel led by an experienced neurologist established the final diagnosis in accordance with the criteria of the Diagnostic and Statistical Manual of Mental Disorders (Third Edition, Revised) for dementia. Dementia subtype was allocated using the National Institute of Neurological Disorders and Stroke‐Alzheimer Disease and Related Disorders (NINCDS‐ADRDA) criteria for Alzheimer's disease,^31^ the[Fig alz13496-fig-0001], [Fig alz13496-fig-0002] National Institute of Neurological Disorders and Stroke‐Association Internationale pour la Recherche et l'Enseignement en Neurosciences (NINDS‐AIREN) criteria for vascular dementia,^32^ the Dementia with Lewy Bodies (DLB) Consortium criteria for dementia with Lewy bodies,^33^ the International Behavioural Variant Frontotemporal Dementia Criteria Consortium (FTDC) criteria for frontotemporal dementia,^34^ and the European Community Concerted Action on the Epidemiology of Parkinson's disease (EUROPARKINSON) criteria for Parkinson's disease (when associated with dementia).^35^, ^36^

### 2.4 Assessment of stroke

Participants were screened for stroke at baseline and afterwards according to a previously reported protocol.^37^ Briefly, screening for stroke took place at study entry and at follow‐up visits via an interview with a trained physician. Additional information on stroke incidence was gathered through continuous linkage with participants’ medical records, including neuroimaging if available. All strokes were reviewed by research physicians and verified by an experienced stroke neurologist. Stroke was defined based on the World Health Organization (WHO) criteria as a syndrome of rapidly developing clinical signs of focal (or global) disturbance of cerebral function, with symptoms lasting ≥24 h or leading to death, with no apparent cause other than of vascular origin.

### 2.5 Assessment of cerebral small vessel disease and subcortical brain structures

Brain MRI scans were obtained with a 1.5‐tesla MRI scanner with a dedicated 8‐channel head coil (General Electric Healthcare, Milwaukee, WI, USA). The scanning protocol has been described in detail previously.^38^ All MRI assessments were performed blinded for calcification scores.

As measures of cSVD we assessed white matter hyperintensity (WMH) volume and the presence of microbleeds and of lacunar infarcts. A previously described, a validated automated tissue‐classification technique was used to quantify WMH volume in milliliters, using fluid‐attenuated inversion recovery (FLAIR) sequences and an atlas‐based k‐nearest‐neighbor classifier.^39^ All segmentations were inspected visually by trained raters and manually corrected when necessary. Lacunar infarcts were rated on FLAIR, proton density–weighted, and T1‐weighted sequences, and were defined as focal lesions ≥3 mm and <15 mm with tissue loss and same signal intensity as cerebrospinal fluid on all sequences and a hyperintense rim on FLAIR.^40^ Microbleeds were rated on T2*‐weighted gradient recalled echo sequences and were defined as focal round to ovoid lesions <10 mm with low signal intensity.^40^

Subcortical brain volumes were assessed on T1‐weighted images using FreeSurfer (version 6.0). We assessed the amygdala, thalamus, and hippocampus, because incorporation of these structures allowed for a discrimination between anterior and posterior cerebral vascular territories, as the amygdala is vascularized predominantly by the anterior circulation, and the thalamus and hippocampus predominantly by the posterior brain circulation.^6^, ^7^ The volume of each structure was calculated as the mean volume of the left and right structures. An automated quality metric was obtained for each scan that was used to identify images of low quality. In addition, 200 randomly selected scans were visually rated for quality assessment.^7^ Location‐specific information on measures of cSVD and cortical brain volumes was not readily available.

### 2.6 Assessment of covariables

Covariables were selected based on previous literature on their link with dementia^28^ and intracranial arteriosclerosis.^19^ In addition to age and sex, we included obesity, diabetes, hypertension, hypercholesterolemia, smoking, excessive alcohol use, education level, apolipoprotein E (*APOE*) genotype, and extracranial carotid artery calcification (ECAC) in analyses. Specifically, ECAC was included as it is related to intracranial calcifications^9^ and is a strong risk factor for dementia.^2^ We defined obesity as a body mass index of ≥30 kg/m^2^, diabetes as a fasting plasma glucose ≥7.0 mmol/L (126.0 mg/dL) and/or the use of antidiabetic medication, hypertension as a systolic blood pressure ≥140 mmHg and/or a diastolic blood pressure ≥90 mmHg and/or the use of blood pressure‐lowering medication, and hypercholesterolemia as a serum total cholesterol of ≥6.2 mmol/L (≥240.0 mg/dL) and/or the use of lipid‐lowering medication. Smoking status was categorized into current, former, and never smokers. Excessive alcohol use was defined as > 20 g/day. Education level was categorized into low (primary education, lower/intermediate general education, lower vocational education), intermediate (higher general education, intermediate vocational education), or high education (university). In 135 participants with missing *APOE* genotype from blood sampling, the genotype was determined by genetic imputation (Illumina 610K and 660K chip; imputation with Haplotype Reference Consortium reference panel [v1.0] with Minimac 3). *APOE* genotype was dichotomized into carriers and non‐carriers of the ε4 risk allele. ECAC was assessed on the same MDCT scanners that were used for intracranial arteriosclerosis assessment, using commercially available software (Syngo Calcium Scoring, Siemens, Germany).^2^

### 2.7 Statistical analysis

To deal with the right‐skewed distributions of ICAC and VBAC volumes, we performed a natural log transformation to all volumes, after adding one mm^3^ to nontransformed values to deal with calcium volumes of zero. Then we analyzed the association between intracranial arteriosclerosis and dementia in all 2339 participants of our main sample using the following strategy. First, we calculated the hazard ratios (HRs) for the presence, volume, and volume tertiles of ICAC and VBAC associated with dementia using Cox models. All Cox models included follow‐up time as timescale, starting from the MDCT scan until the time of incident dementia, death, loss to follow‐up, or January 1, 2020. Presence and volume of calcification were assessed in all persons, whereas volume tertiles were assessed only in persons with prevalent calcification (limited VBAC group size restricted the possibility to use quartiles). Model 1 was adjusted for age and sex. Model 2 was additionally adjusted for smoking, hypercholesterolemia, hypertension, diabetes, obesity, excessive alcohol use, education level, *APOE* ε4 carrier status, and ECAC volume. Dichotomized risk factors were used to maximize analytical power while allowing for the adjustment of many potential confounders, to facilitate comparison with prior studies,^2^, ^41^ and because these best reflect risk factors as used in the clinic. We verified the proportional hazards assumption by visual assessment of log–log survival plots. Second, we assessed the HR of the presence, volume, and volume tertiles of the atherosclerotic and IEL subtypes of ICAC associated with dementia using Cox models with similar adjustments. Presence and volume included persons with a subtype and those without ICAC, whereas analyses on volume tertiles included only persons with a particular subtype.

Then to further explore mechanism underlying the impact of arteriosclerosis on dementia, we performed causal mediation analyses in our subsample of 1478 participants who had undergone an MRI scan of the head. We assessed the direct impact of volumes of ICAC, ICAC subtypes, and VBAC on dementia, and the indirect impact occurring through cSVD (WMH volume, presence of microbleeds, presence of lacunes) and subcortical brain structure volumes (thalamus, hippocampus, amygdala) using regression‐based causal mediation analyses (closed form parameter estimation and delta method standard errors).^42^ WMH volume was natural‐log transformed (after adding 1 μL to deal with zeroes) to deal with the right skewed distribution. We included all covariables mentioned previously in model 2 of the Cox models as exposure‐outcome, exposure‐mediator, and mediator‐outcome confounders, in addition to the total intracranial volume and the time difference in between the computed tomography (CT) and MRI scan. We used linear (volumes of WMHs, thalamus, hippocampus, and amygdala) or logistic regression (presence of microbleeds and of lacunar infarcts) as mediator models. Adding exposure‐mediator interaction to models had minimal impact on estimates and revealed non‐significant interaction terms, so no exposure‐mediator interaction was modeled.^42^

Finally, we performed three sensitivity analyses. First, we repeated all Cox models classifying AD as event instead of any dementia. Second, although the a priori probability of a mediating effect by stroke was limited due to the small amount of strokes that occurred before dementia (*n* = 22), we attempted to verify this by repeating analyses after excluding those persons. Third, we repeated Cox analyses with an additional quadratic term for age added to models to assess residual confounding. Fourth, we repeated Cox analyses using continuous instead of dichotomized covariables, for example using systolic blood pressure and antihypertensive medication use instead of the dichotomous hypertension variable.

We accounted for missing values using multiple imputation (Markov Chain Monte Carlo method, maximum proportion of missing values 3%). Missingness was not related to incident dementia for any variable. All analyses were performed using R 4.2.0 (R Foundation for Statistical Computing, Vienna, Austria) and RStudio (RStudio: Integrated Development for R, Boston, United States). Statistical significance was set to a *p*‐value < 0.05.

## 3 RESULTS

### 3.1 Characteristics of the study population

Table 1 shows the characteristics of the study population at baseline. The mean age was 69.5 years, and 52.2% of the participants were women. The prevalence of ICAC was 81.4% and VBAC was prevalent in 20.1% of participants. Of all persons with ICAC, 39.2% showed a predominant atherosclerotic subtype and 48.0% an IEL subtype, whereas both were present equally in 12.8% of participants with ICAC. During a median follow‐up of 13.4 years (27,231.2 person years), 282 participants developed dementia, of which 217 (77.0%) were classified as clinical AD. In total, 214 persons had a stroke, of which 22 occurred before a dementia diagnosis. In addition, 617 persons died during follow‐up. Table S1 shows the characteristics of participants included in the mediation analyses.

**TABLE 1:** Baseline characteristics of the study population.

Characteristic	Total
Sample size, *N*	2339
Age, years	69.5 (6.7)
Sex, female	1221 (52.2)
Body mass index, kg/m^2^	27.7 (4.0)
Serum glucose, mmol/L	5.4 (5.1–5.9)
Glucose‐lowering medication use	146 (6.2)
Systolic blood pressure, mmHg	146.6 (20.0)
Diastolic blood pressure, mmHg	80.2 (10.8)
Blood pressure–lowering medication use	927 (39.6)
Total cholesterol, mmol/L	5.7 (1.0)
HDL cholesterol, mmol/L	1.4 (0.4)
Lipid‐lowering medication use	560 (23.9)
Smoking status	
Current	296 (12.7)
Former	1325 (56.6)
Never	718 (30.7)
Excessive alcohol use	585 (25.0)
Education level	
High	448 (19.2)
Intermediate	730 (31.2)
Low	1161 (49.6)
Mini‐Mental State Examination score	28 (28–29)
*APOE* ε4 carrier	620 (26.5)
ICAC, presence	1904 (81.4)
Atherosclerotic subtype	746 (31.9)
IEL subtype	913 (39.0)
Mixed subtype	245 (10.5)
VBAC, presence	469 (20.1)

*Note*. Values are absolute numbers (percentage) for categorical variables, mean (SD) for normally distributed continuous variables, and median (25th–75th percentiles) for non‐normally distributed variables.

Abbreviations: *APOE*, apolipoprotein E; HDL, high‐density lipoprotein; ICAC, intracranial carotid artery calcification; IEL, internal elastic lamina; VBAC, vertebrobasilar artery calcification.

### 3.2 Intracranial arteriosclerosis and the risk of dementia

Table 2 shows that the presence of ICAC (HR: 1.53, 95% confidence interval [CI]: 1.00–2.32) and ICAC volume (HR per SD increase: 1.19, 95% CI: 1.01–1.40) increased dementia risk, after adjustment for confounders and for extracranial arteriosclerosis. Both the atherosclerotic and the IEL subtype of ICAC were associated with an increased risk of dementia (Table 3). Especially severe IEL ICAC conferred a large dementia risk (third tertile of calcification volume vs first—HR 2.22, 95% CI: 1.24–3.97). For VBAC, particularly severe calcifications increased dementia risk (HR for third tertile of calcification volume compared to first—1.89, 95% CI: 1.00–3.59; Table 2).

**TABLE 2:** Intracranial arteriosclerosis and the risk of dementia.

			Hazard ratio for dementia (95% CI)	
Calcification		*N*/*n*	Model 1	Model 2
ICAC	Presence	281/2339	1.58 (1.08–2.30)	1.53 (1.00–2.32)
	Per SD	281/2339	1.26 (1.10–1.44)	1.19 (1.01–1.40)
	Q1	52/635	Reference	Reference
	Q2	93/635	1.40 (0.99–1.99)	1.20 (0.83–1.73)
	Q3	106/634	1.53 (1.07–2.19)	1.44 (0.79–2.64)
VBAC	Presence	281/2339	1.09 (0.82–1.44)	1.15 (0.86–1.54)
	Per SD	281/2339	1.10 (0.99–1.23)	1.10 (0.98–1.24)
	Q1	20/157	Reference	Reference
	Q2	20/156	1.55 (0.83–2.91)	1.26 (0.64–2.50)
	Q3	29/156	1.88 (1.07–3.31)	1.89 (1.00–3.59)

*Note*. Hazard ratios (95% CI) of the presence, volume, and volume tertiles of intracranial calcifications associated with dementia. Presence and volume were assessed in all participants. Volume tertiles were assessed in persons with ICAC (*n* = 1904) or VBAC (*n* = 469). Model 1 was age and sex adjusted. Model 2 was additionally adjusted for smoking, hypercholesterolemia, hypertension, diabetes, obesity, excessive alcohol use, education level, *APOE* ε4 carrier status, and ECAC.

Abbreviations: *APOE*, apolipoprotein E; CI, confidence interval; ECAC, extracranial carotid artery calcification.; ICAC, intracranial carotid artery calcification; *N*, number of dementia cases; *n*, number of persons at risk; Q, tertile; SD, standard deviation; VBAC, vertebrobasilar artery calcification.

**TABLE 3:** Subtypes of intracranial carotid artery arteriosclerosis and the risk of dementia.

			Hazard ratio for dementia (95% CI)	
ICAC subtype		*N*/*n*	Model 1	Model 2
Atherosclerotic	Presence	119/1181	1.56 (1.03–2.35)	1.50 (0.98–2.29)
	Per SD	88/1181	1.33 (1.08–1.63)	1.28 (1.03–1.59)
	Q1	20/249	Reference	Reference
	Q2	24/249	1.04 (0.56–1.91)	1.22 (0.65–2.29)
	Q3	44/248	1.41 (0.80–2.48)	1.37 (0.76–2.46)
IEL	Presence	163/1384	1.59 (1.06–2.38)	1.43 (0.94–2.17)
	Per SD	163/1384	1.26 (1.08–1.47)	1.19 (1.01–1.39)
	Q1	24/305	Reference	Reference
	Q2	51/304	1.80 (1.09–2.98)	1.73 (1.02–2.94)
	Q3	57/304	2.11 (1.26–3.53)	2.22 (1.24–3.97)

*Note*. Hazard ratios (95% CIs) of the presence, volume, and volume tertiles of ICAC subtypes associated with dementia. Presence and volume were assessed in participants with the denoted subtype and those without ICAC (*n* = 435). Volume tertiles were assessed in persons with atherosclerotic ICAC (*n* = 746) or IEL ICAC (*n* = 913). Model 1 was age and sex adjusted. Model 2 was additionally adjusted for smoking, hypercholesterolemia, hypertension, diabetes, obesity, excessive alcohol use, education level, *APOE* ε4 carrier status, and ECAC.

Abbreviations: *APOE*, apolipoprotein E; CI, confidence interval; ECAC, extracranial carotid artery calcification; ICAC, intracranial carotid; IEL, internal elastic lamina; *N*, number of dementia cases; *n*, number of persons at risk; Q, tertile; SD, standard deviation.

### 3.3 Direct and indirect effects on dementia risks

In causal mediation analyses, the effects of ICAC and VBAC on dementia were independent of subcortical brain structure volumes (Table 4). Rather, the effects of both ICAC and VBAC on dementia were partly mediated through increased WMH volumes (percentage mediated for ICAC: 13% and VBAC: 24%, Table 4). For ICAC specifically, the indirect effect through WMH volume occurred only for IEL ICAC (HR: 1.03, 95% CI: 1.00–1.06, percentage mediated: 14%) and not for atherosclerotic ICAC (HR: 1.01, 95% CI: 0.98–1.04). No mediation occurred through presence of lacunar infarcts or microbleeds.

**TABLE 4:** The direct effects of intracranial arteriosclerosis on dementia and indirect effects through cerebral small vessel disease and subcortical brain structure volumes.

Calcification (per SD)		Mediated by WMH volume (per SD)	Mediated by presence of lacunar infarcts	Mediated by presence of microbleeds
ICAC	Direct effect	1.33 (1.08–1.63)	1.37 (1.12–1.68)	1.38 (1.13–1.69)
	Indirect effect	1.04 (1.01–1.07)	1.02 (1.00–1.04)	1.01 (1.00–1.03)
	% mediated	13%	NS	NS
VBAC	Direct effect	1.16 (0.99–1.36)	1.25 (1.08–1.44)	1.25 (1.08–1.45)
	Indirect effect	1.04 (1.01–1.07)	1.00 (0.99–1.01)	1.01 (1.00–1.03)
	% mediated	24%	NS	NS

Calcification (per SD)		Mediated by thalamus volume (per SD)	Mediated by hippocampus volume (per SD)	Mediated by amygdala volume (per SD)
ICAC	Direct effect	1.34 (1.07–1.68)	1.32 (1.06–1.64)	1.31 (1.05–1.64)
	Indirect effect	1.00 (0.99–1.02)	1.00 (0.97–1.03)	1.00 (0.97–1.03)
	% mediated	NS	NS	NS
VBAC	Direct effect	1.23 (1.03–1.46)	1.16 (0.98–1.38)	1.20 (1.01–1.42)
	Indirect effect	1.00 (0.99–1.01)	1.02 (0.99–1.05)	1.00 (0.99–1.04)
	% mediated	NS	NS	NS

*Note*. Hazard ratios (95% CIs) of the direct effects of intracranial calcification volume on dementia and the indirect effects through cerebral small vessel disease (WMH volume, lacunar infarcts, microbleeds) and subcortical brain structure volumes assessed in the subsample of participants who underwent an MRI scan (*n* = 1478). If the indirect effect was significant (*p* < 0.05) the percentage mediated is denoted. All models were adjusted for age, sex, hypercholesterolemia, hypertension, diabetes, obesity, excessive alcohol use, education level, *APOE* ε4 carrier status, ECAC, the time difference in between the MDCT and MRI scan, and total intracranial volume.

Abbreviations: APOE, apolipoprotein E; CI, confidence interval; ECAC, extracranial carotid artery calcification; ICAC, intracranial carotid artery calcification; MDCT, multidetector computed tomography; MRI, magnetic resonance imaging.; NS, not significant; SD, standard deviation; VBAC, vertebrobasilar artery calcification; WMH, white matter hyperintensity.

### 3.4 Sensitivity analyses

The associations of ICAC, VBAC, and ICAC subtypes with the risk of clinical AD were similar to the risks of all‐cause dementia (‐Table S2). Excluding participants who had a stroke (*n* = 22) did not change the results. Adding a quadratic term for age to models did not materially impact findings. Similarly, interpretation did not change when using models including continuous covariables and medication use instead of dichotomized covariables (Table S3).

## 4 DISCUSSION

We found that intracranial arteriosclerosis was linked to an increased risk of dementia, and that part of this link could be explained by mediation through increased WMH volume.

We found a clear association between anterior cerebral arteriosclerosis, namely, that of the intracranial carotid artery, and an increased dementia risk. Anterior cerebral arteriosclerosis starts developing relatively early in life and progresses to affect virtually all individuals aged 70 years old and over.^43^ Given its ubiquity and considerable impact on dementia risk, successful reduction of anterior cerebral arteriosclerosis might have a substantial impact on the global burden of dementia. In addition to this finding, we found that severe arteriosclerosis of the posterior cerebral circulation, namely, the vertebrobasilar arteries, also considerably increased the risk of dementia. Even though posterior cerebral arteriosclerosis is less prevalent than its anterior counterpart, given the relatively large risk that it confers it might still contribute to a substantial fraction of dementia risk, especially in populations with a large burden of intracranial arteriosclerosis (e.g. stroke patients).^19^ As the posterior cerebral circulation provides vascularization to several brain structures that are important in dementia etiology (e.g., thalamus,^7^ hippocampus,^7^ periventricular white matter,^44^ and cerebellum^45^), this finding raised the question of whether the effects of posterior cerebral arteriosclerosis are location specific, or whether they reflect a general effect of intracranial arteriosclerosis (as most persons with extensive posterior cerebral arteriosclerosis also suffer from anterior cerebral arteriosclerosis.^6^ By assessing mediation through subcortical brain structure volumes, we explored these potential distinct effects based on the supplying vascular territory (i.e. the anterior vs posterior cerebral circulation), but these analyses did not reveal any mediating effects. We did find, however, that a particularly large part of the effect of posterior cerebral arteriosclerosis on dementia occurred via increased WMHs, which, as WMHs occur primarily in regions that are vascularized by the posterior circulation, might reflect a location‐specific mechanism.^46^ Future studies might elaborate on the location‐specific effects of both posterior and anterior cerebral arteriosclerosis on the brain and dementia.

Interestingly, we found that both the atherosclerotic and the IEL subtype of anterior cerebral arteriosclerosis increased dementia risk, particularly severe IEL arteriosclerosis. We can use these findings to further elaborate upon the potential underlying mechanisms. The IEL subtype of arteriosclerosis is elongated and circular and is thought to contribute primarily to arterial stiffening. The atherosclerotic subtype is short and thick, occluding the vessel lumen, potentially leading to cerebral hypoperfusion, another important risk factor for dementia.^47^ In corroboration of these proposed effects, we found that the effects of only the IEL subtype were mediated through WMHs, which are thought to be caused by arterial stiffening–induced hypertensive stresses rather than hypoperfusion (although there is debate on the exact mechanisms).^48^ These findings indicate that the IEL calcifications of the carotid siphon stiffen the artery, leading to increased WMLs and to dementia. Secondary to these effects, dysfunction of the blood–brain barrier (BBB) might also play a role: vascular injury due to cerebral hypoperfusion or arterial stiffness might impair BBB integrity, which has been linked to dementia through reduced clearance of neurotoxic materials from the brain.^49^

### 4.1 Limitations

Strengths of the current study include the unique population‐based setting, the extensive follow‐up time, the comprehensive assessment of intracranial arteriosclerosis (including anterior, posterior, and subtypes of intracranial arteriosclerosis), and the thorough consideration of potential confounders and mediators in analyses. There are also important limitations to consider. As most individuals who developed dementia in our study had not previously had a stroke (in line with previous findings^50^), limited power restricted formal assessment of a potential mediating effect by stroke. Regarding external validity, our sample almost exclusively involves persons of Caucasian ancestry. As the burden of intracranial atherosclerosis is dependent on ancestry, it is conceivable the same holds true for intracranial arteriosclerosis.^51^ Although we expect the hemodynamic consequences of arteriosclerosis to be consistent across ancestries, such differences may render one location or subtype of intracranial arteriosclerosis more (or less) important in terms of its impact on dementia risk in a particular population. Then there may be unmeasured confounding of the exposure‐outcome and/or mediator‐outcome relationships. Assuming a confounder that is either positively or negatively related to exposure, mediator, and outcome, this might have led to an overestimation (exposure‐outcome confounder) or underestimation (mediator‐outcome confounder) of results.^52^ Next, we did not have biomarker information or post‐mortem assessment available to confirm etiological subtypes of dementia diagnoses. Subsequent misclassifications, likely to have occurred equally among persons with and without intracranial arteriosclerosis, could have led to an underestimation of the impact of arteriosclerosis on dementia. Nonetheless, the majority of older patients with dementia have a combination of different pathologies that preclude a single etiological diagnosis and are, therefore, best captured with an all‐cause dementia diagnosis.^53^

### 4.2 Conclusion

Our findings indicate that intracranial arteriosclerosis plays an important role in the etiology of dementia. Several potential underlying mechanisms have been explored, which indicate that the different subtypes of arteriosclerosis distinctly impact the brain and are, therefore, important factors to consider in the etiology of dementia. Because several therapeutic options are under investigation aimed at reducing the burden of arteriosclerosis, our findings emphasize the potential of intracranial arteriosclerosis as an important avenue for dementia prevention.^21^

## CONFLICT OF INTEREST STATEMENT

The authors declare no conflicts of interest.Author disclosures are available in the supporting information.

## CONSENT STATEMENT

The Rotterdam Study has been approved by the medical ethics review committee of the Erasmus Medical Center in Rotterdam (number MEC02.1015) and has been entered into the WHO International Clinical Trials Registry Platform (number NTR6831). All participants provided written informed consent for participation.

## Supporting information

Supporting Information

Supporting Information
