# Supplementary material for: Intracranial arteriosclerosis and the risk of dementia: A population‐based cohort study
Source: Alzheimers Dement. 2023 Oct 9;20(2):869–79. doi: 10.1002/alz.13496 (PMC10916985; doi:10.1002/alz.13496)
Supplement: Supplementary file 2 — Supporting Information [file ALZ-20-869-s001.docx]

Supplementary Material

**Supplementary Table 1**. Characteristics of the subsample with a brain MRI for mediation analyses.

| **Characteristic** | **Total** |
| --- | --- |
| N | 1478 |
| Age (years) | 68.0 (5.7) |
| Sex (female) | 758 (51.3%) |
| ICAC presence | 1173 (79.4%) |
| VBAC presence | 253 (17.1%) |
| Time in between MDCT and MRI scans (years) | 1.4 (0.6-6.1) |
| WMH volume (mL) | 4.4 (2.5-8.7) |
| Lacunar infarct presence | 135 (9.1%) |
| Microbleed presence | 367 (24.8%) |
| Thalamus volume (mL) | 6.4 (0.6) |
| Hippocampus volume (mL) | 3.8 (0.4) |
| Amygdala volume (mL) | 1.4 (0.2) |

Characteristics are presented as number (percentage) for categorical variables, mean (standard deviation) for normally distributed continuous variables, and median (25^th^-75^th^ percentiles) for non-normally distributed variables.

Abbreviations: ICAC, intracranial carotid artery calcification; VBAC, vertebrobasilar artery calcification; MDCT, multidetector computed tomography; MRI, magnetic resonance imaging; WMH, white matter hyperintensity.

**Supplementary Table 2**. Intracranial arteriosclerosis and the risk of clinical Alzheimer’s disease.

| **Calcification** | | **N/n** | **Hazard ratio for Alzheimer’s disease** | |
| --- | --- | --- | --- | --- |
|  |  |  | Model 1 | Model 2 |
| ICAC | Presence | 217/2339 | 1·41 (0·93-2·13) | 1·32 (0·83-2·10) |
|  | Volume (per SD) | 217/2339 | 1·21 (1·03-1·40) | 1·19 (1·00-1·42) |
|  | Q1 | 40/635 | Reference | Reference |
|  | Q2 | 69/635 | 1·35 (0·90-2·02) | 1·19 (0·78-1·80) |
|  | Q3 | 82/634 | 1·45 (0·96-2·19) | 1·38 (0·89-2·14) |
| Atherosclerotic ICAC | Presence | 91/1181 | 1·40 (0·88-2·21) | 1·30 (0·80-2·09) |
|  | Volume (per SD) | 91/1181 | 1·24 (0·98-1·57) | 1·19 (0·93-1·52) |
|  | Q1 | 16/249 | Reference | Reference |
|  | Q2 | 18/249 | 1·02 (0·51-2·02) | 1·24 (0·60-2·57) |
|  | Q3 | 31/248 | 1·29 (0·68-2·45) | 1·29 (0·64-2·58) |
| IEL ICAC | Presence | 129/1384 | 1·38 (0·88-2·16) | 1·18 (0·74-1·89) |
|  | Volume (per SD) | 129/1384 | 1·19 (1·00-1·41) | 1·09 (0·91-1·31) |
|  | Q1 | 16/305 | Reference | Reference |
|  | Q2 | 42/304 | 2·11 (1·17-3·80) | 1·78 (0·96-3·32) |
|  | Q3 | 45/304 | 2·40 (1·31-4·42) | 2·46 (1·25-4·86) |
| VBAC | Presence | 217/2339 | 0·96 (0·69-1·33) | 1·06 (0·75-1·48) |
|  | Volume (per SD) | 217/2339 | 1·09 (0·97-1·23) | 1·63 (0·72-3·70) |
|  | Q1 | 12/157 | Reference | Reference |
|  | Q2 | 15/156 | 2·13 (0·99-4·59) | 1·63 (0·72-3·70) |
|  | Q3 | 22/156 | 2·40 (1·19-4·82) | 2·82 (1·29-6·18) |

Hazard ratios (95% CI) of the presence, volume, and volume tertiles of intracranial calcifications associated with Alzheimer’s disease. For ICAC and VBAC, presence and volume were assessed in all participants and volume tertiles were assessed in persons with ICAC (n=1904) or VBAC (n=469). For ICAC subtypes, presence and volume were assessed in participants with the denoted subtype and those without ICAC (n=435) and volume tertiles were assessed in persons with atherosclerotic ICAC (n=746) or IEL ICAC (n=913). Model 1 was age and sex adjusted. Model 2 was additionally adjusted for smoking, hypercholesterolemia, hypertension, diabetes, obesity, excessive alcohol use, education level, APOE ε4 carrier status, and ECAC.

Abbreviations: N, number of dementia cases; n, number of persons at risk; CI, confidence interval; ICAC, intracranial carotid artery calcification; VBAC, vertebrobasilar artery calcification; SD, standard deviation; Q, tertile; APOE, apolipoprotein E; ECAC, extracranial carotid artery calcification.

**Supplementary Table 3**. Intracranial arteriosclerosis and the risk of dementia using continuous covariables and medication use instead of dichotomized covariables.

| **Calcification** | | **N/n** | **Hazard ratio for dementia** |
| --- | --- | --- | --- |
| ICAC | Presence | 281/2339 | 1·32 (0·83-2·10) |
|  | Volume (per SD) | 281/2339 | 1·19 (1·00-1·42) |
|  | Q1 | 52/635 | Reference |
|  | Q2 | 93/635 | 1·19 (0·78-1·80) |
|  | Q3 | 106/634 | 1·38 (0·89-2·14) |
| Atherosclerotic ICAC | Presence | 119/1181 | 1·30 (0·80-2·09) |
|  | Volume (per SD) | 88/1181 | 1·19 (0·93-1·52) |
|  | Q1 | 20/249 | Reference |
|  | Q2 | 24/249 | 1·24 (0·60-2·57) |
|  | Q3 | 44/248 | 1·29 (0·64-2·58) |
| IEL ICAC | Presence | 163/1384 | 1·18 (0·74-1·89) |
|  | Volume (per SD) | 163/1384 | 1·09 (0·91-1·31) |
|  | Q1 | 24/305 | Reference |
|  | Q2 | 51/304 | 1·78 (0·96-3·32) |
|  | Q3 | 57/304 | 2·46 (1·25-4·86) |
| VBAC | Presence | 281/2339 | 1·06 (0·75-1·48) |
|  | Volume (per SD) | 281/2339 | 1·63 (0·72-3·70) |
|  | Q1 | 20/157 | Reference |
|  | Q2 | 20/156 | 1·63 (0·72-3·70) |
|  | Q3 | 29/156 | 2·82 (1·29-6·18) |

Hazard ratios (95% CI) of the presence, volume, and volume tertiles of intracranial calcifications associated with dementia. For ICAC and VBAC, presence and volume were assessed in all participants and volume tertiles were assessed in persons with ICAC (n=1904) or VBAC (n=469). For ICAC subtypes, presence and volume were assessed in participants with the denoted subtype and those without ICAC (n=435) and volume tertiles were assessed in persons with atherosclerotic ICAC (n=746) or IEL ICAC (n=913). All associations were additionally adjusted for BMI, plasma glucose (mmol/L), glucose lowering medication use, systolic blood pressure (mmHg), blood pressure lowering medication use, total cholesterol (mmol/L), lipid lowering medication use, smoking, education level, APOE ε4 carrier status, and ECAC volume.

Abbreviations: N, number of dementia cases; n, number of persons at risk; CI, confidence interval; ICAC, intracranial carotid artery calcification; VBAC, vertebrobasilar artery calcification; SD, standard deviation; Q, tertile; APOE, apolipoprotein E; ECAC, extracranial carotid artery calcification.
